# Brain Localization and Neurotoxicity Evaluation of Polysorbate 80-Modified Chitosan Nanoparticles in Rats

**DOI:** 10.1371/journal.pone.0134722

**Published:** 2015-08-06

**Authors:** Zhong-Yue Yuan, Yu-Lan Hu, Jian-Qing Gao

**Affiliations:** Institute of Pharmaceutics, College of Pharmaceutical Sciences, Zhejiang University, Hangzhou, 310058, P. R. China; National Institute of Health (NIH), UNITED STATES

## Abstract

The toxicity evaluation of inorganic nanoparticles has been reported by an increasing number of studies, but toxicity studies concerned with biodegradable nanoparticles, especially the neurotoxicity evaluation, are still limited. For example, the potential neurotoxicity of Polysorbate 80-modified chitosan nanoparticles (Tween 80-modified chitosan nanoparticles, TmCS-NPs), one of the most widely used brain targeting vehicles, remains unknown. In the present study, TmCS-NPs with a particle size of 240 nm were firstly prepared by ionic cross-linking of chitosan with tripolyphosphate. Then, these TmCS-NPs were demonstrated to be entered into the brain and specially deposited in the frontal cortex and cerebellum after systemic injection. Moreover, the concentration of TmCS-NPs in these two regions was found to decrease over time. Although no obvious changes were observed for oxidative stress in the *in vivo* rat model, the body weight was found to remarkably decreased in a dose-dependent manner after exposure to TmCS-NPs for seven days. Besides, apoptosis and necrosis of neurons, slight inflammatory response in the frontal cortex, and decrease of GFAP expression in the cerebellum were also detected in mouse injected with TmCS-NPs. This study is the first report on the sub-brain biodistribution and neurotoxicity studies of TmCS-NPs. Our results provide new insights into the toxicity evaluation of nanoparticles and our findings would help contribute to a better understanding of the neurotoxicity of biodegradable nanomaterials used in pharmaceutics.

## Introduction

Because of the unique physicochemical properties, nanomaterials are now becoming more and more popular and have a wide application in many fields, such as in biology, medicine, and biochemical engineering[1–3]. Thus, the potential toxicity of nanomaterials should be extensively examined to ensure their safety. To date, studies on nanotoxicity are primarily limited to the toxicity of inorganic nanoparticles, such as carbon nanotubes[4], zinc oxide nanoparticles[5], nanoscale silver clusters[6], and titanium dioxide[7] Toxicity studies of biodegradable nanoparticles, such as chitosan nanoparticles, are still few. Because of their several advantages such as better stability, biocompatibility, biodegradability, as well as simple preparation methods, chitosan nanoparticles (CS-NPs) have been developed for controlled drug delivery, tissue engineering, nonviral gene delivery, as well as vaccine delivery[8, 9]. Several studies have demonstrated that CS-NPs elicited cytotoxicity in tumor cells. For example, the proliferation of a tumor cell line has been found to be inhibited dose-dependently by 65 nm CS-NPs[10]. Further, CS-NPs also exhibited a dose- and time- dependent growth anticancer activity *in vitro* and *in vivo*[11]. Recently, Park et al.[12] reported that exposure of mouse embryos to 100 nm CS-NPs during *in vitro* culture induced blastocyst complications and downregulated the expression of trophectoderm-associated and pluripotent marker genes. Additionally, our previous study also demonstrated that CS-NPs with a particle size of 200 nm elicited dose-dependent decrease on the hatching and survival rates in a zebrafish embryo model[13]. A bent spine in the zebrafish was also observed, which was proposed to be caused by potential neurotoxicity of the CS-NPs. Therefore, the toxicity of those biodegradable nanoparticles could not be ignored.

CS-NPs need to be modified to transport drugs into the brain. Polysorbate 80-modified CS-NPs (TmCS-NPs) have been demonstrated to facilitate the transport of drugs to the brain, hence, it was used as a drug delivery vehicle for brain targeting[14, 15]. Trapani et al.[14] suggested that TmCS-NPs with a particle size ranging from 200 nm to 300 nm could act as brain targeting carriers. Thus, our studies were mainly focused on the neurotoxicity of TmCS-NPs with a particle size of 240 nm. TmCS-NPs have been used as a vehicle for the treatment of brain disorders[14, 16] and brain diseases are usually treated clinically by multiple dose administration. Thus, the multiple-dose intravenous toxicity studies were performed to assess the neurotoxicity of TmCS-NPs.

Brain is vulnerable to oxidative damage because of its high content of easily peroxidizable unsaturated fatty acids, high oxygen consumption rate, and relative paucity of antioxidant enzymes compared with other organs[17]. Nanoparticles may enter into the brain and cause brain injury because of their unique physicochemical properties (e.g., large surface area). Thus, the toxicity of nanocarriers, specifically those for the brain, should be carefully considered. Brain is composed primarily of two cell types, namely, neurons and glial cells. Thus, in this study, the expression of NeuN and GFAP was investigated to estimate the changes of neurons and glial cells, respectively. Oxidative stress reaction has been identified as the common mechanism[18–21]. Oxidative stress may result in inflammation and consequent damage to DNA and proteins, which are involved in brain damage[22]. Thus, oxidative stress nanotoxicity may be the mechanism.

In the present study, TmCS-NPs were prepared based on the ionic gelation of chitosan with tripolyphosphate (TPP). A fluorescence marker, rhodamine B isothiocyanate (RBITC), was adopted to label the chitosan to detect the distribution, localization, and accumulation of the TmCS-NPs in the brain after intravenous injection. Then, rats were exposed to three TmCS-NP concentrations. Consequently, the potential particle induced injuries and function changes in the brain were evaluated. Oxidative stress, one of the best-known factors for apoptosis, was assessed by measuring the activity of glutathione (GSH) and levels of hydrogen peroxide (H_2_O_2_) and lipid peroxidation (MDA) in the accumulation regions of the brain. Daily body weight of the rats was measured before tail-vein injection. Histopathological analyses of the brain were also performed to assess the tissue morphology. GFAP expression and NeuN expression were investigated to assess the changes of astrocytes and neurons, respectively. To the best of our knowledge, this study is the first to report on the sub-brain distribution and neurotoxicity study of TmCS-NPs. Results from this study are expected to provide more insights into the toxicity evaluation of nanoparticles and offer a better understanding of the neurotoxicity of biodegradable nanocarriers used as drug delivery vehicles.

## Materials and Methods

### Animals and Ethics Statements

Seven-week-old Sprague-Dawley (SD) male rats (180 g to 220 g) were supplied by Zhejiang University Experimental Animal Center, China. All animals were maintained at 25°C ± 1°C, with free access to standard diet and drinking water. All experimental procedures were carried out in strict accordance with the recommendations in the Guide for the Care and Use of Laboratory Animals of the National Institutes of Health. The protocol was approved by the Committee on the Ethics of Animal Experiments of Zhejiang University (Animal Experimentation Ethics Approval No.:Zju2012-0052). All surgery was performed under chloral hydrate anesthesia, and all efforts were made to minimize suffering.

### Reagents

Chitosan (85% deacetylation, molecular weight 10^5^ Da) was purchased from Zhejiang Jinke Biochemistry (Zhejiang, China). Rhodamine B isothiocyante (RBITC) was obtained from Sigma-Aldrich (USA). Sodium tripolyphosphate (TPP) was purchased from Shijiazhuang Shinearly Chemicals (China). Polysorbate 80 was obtained from Sinopharm Chemical Reagent (China). The following kits were purchased from Beyotime (Haimen, China): H_2_O_2_ assay kit (S0038), total glutathione assay kit (S0052), and lipid peroxidation MDA assay kit (S0131). Anti-NeuN antibody (MAB377) was purchased from Millipore (MA, USA). Rabbit anti-GFAP was obtained from Wuhan Boster Company (Wuhan, China).

### Fluorescence labeling of chitosan with RBITC

The synthesis of RBITC-labeled chitosan was based on the reaction between the primary amino group of chitosan and the isothiocyanate group of RBITC. Chitosan (0.5 g) was dissolved in 100 mL 2% (v/v) acetic acid solution under magnetic stirring for 30 min. The pH of the solution was adjusted to 7.5 with 1 mol/L NaOH. RBITC (1 mg) in 1 mL DMSO was added to the chitosan solution under stirring. After 1 h reaction in the dark at 40°C, followed by overnight reaction at room temperature, the reaction solution was dialyzed with a dialysis tube (MW: 8000–14000, supplied by Bejing Solarbio Sience & Technology Co., Ltd) in distilled water to remove the free RBITC. Finally, the RBITC-labeled chitosan was characterized by Fourier-transform infrared (FTIR) spectroscopy (FT/IR-4100; Jasco, Japan).

### Preparation and characterization of the TmCS-NPs and RBITC-labeled TmCS-NPs

TmCS-NPs were prepared by the ionic gelation method adapted from other investigations[23, 24]. Chitosan solution (2.0 mg/mL) was prepared by dissolving the polymer in 2% (v/v) acetic acid solution under magnetic stirring. Then, after the pH adjustment of the chitosan solution to pH 5.5 with 1 mol/mL NaOH, 8 mL TPP solution (1 mg/mL) was added dropwise to the chitosan solution under magnetic stirring (1000 rpm) to form the nanoparticles. After 1 h of reaction, 1 mL of an aqueous solution of the surfactant (1.5 g/mL) was mixed with the nanoparticle solution. The nanosuspension was centrifuged at 18,000 rpm and 4°C for 0.5 h. Afterward, the nanoparticles at the bottom were collected. For the RBITC-labeled TmCS-NPs, RBITC-labeled chitosan was used to prepare the nanoparticles instead of chitosan.

The zeta-potential, particle size, and polydispersity index (PDI) of the nanoparticles were determined by laser diffraction spectrometry (Malvern Zetasizer 3000HS; Malvern, Worcestershire, UK) after dilution of the nanoparticles with distilled water. Transmission electron microscopy (TEM) was performed to characterize the morphology of the NPs.

### Brain localization and accumulation

Information on the biodistribution of the nanoparticles is important in investigating the effects of TmCS-NPs on CNS. RBITC-labeled TmCS-NPs were injected intravenously in rats. Then, rats were sacrificed by perfusion at each time point (0.5, 2, 4, 8, and 24 h) and brains were collected. Subsequently, the fluorescence intensity was measured using *in vivo* imaging system (Maestro, CRI, USA). Finally, the sub-brain regions were divided, and the fluorescence intensity was measured.

### Oxidative-stress-related biomarker assay and body weight change

As the frontal cortex and cerebellum are the major accumulation regions, we focused on the damage occurred in these two regions. The rats were divided into four groups according to the dose of intravenous injection received once a day for 7 d: (1) control, physiological saline (1 mg/kg); (2) low-dose, 3 mg/kg TmCS-NPs; (3) middle-dose, 10 mg/kg TmCS-NPs; and (4) high-dose, 30 mg/kg TmCS-NPs. Six rats per group were sacrificed. Daily body weight was collected before tail-vein injection, which started from the first injection. The tissues of the frontal cortex and cerebellum were harvested, and weighed. GSH activity and the levels of H_2_O_2_ and MDA in these two regions were measured by total GSH, H_2_O_2_, and lipid peroxidation MDA assay kits, respectively. The assays were performed following the manufacturers’ instructions.

### Histopathological examination

After exposure for 7 d, the brains of each group were harvested on day 8, and fixed in 10% formalin solution and dehydrated with ethanol (75%, 85%, 95%, and 100%). Paraffin tissue sections (10 μm) of the frontal cortex and cerebellum were prepared. Sections were stained with hematoxylin and eosin (H&E) and photographed with a polarizing microscope (Nikon, Japan) to evaluate the tissue morphology.

### Immunohistological staining of GFAP and NeuN

Paraffin tissue sections of the frontal cortex and cerebellum were prepared as previously mentioned. Immunohistological staining tests were performed according to standard laboratory procedures. In brief, the sections were deparaffinized in xylol, rehydrated in ethanol, and incubated with 5% bovine serum albumin followed by the rabbit anti-GFAP and anti-NeuN antibody. The sections were incubated with horseradish peroxidase (HRP)-labeled secondary antibody, and then incubated with diaminobenzidine (DAB) substrate. Finally, the expression and positive rate of GFAP and NeuN were observed with a polarizing microscope (Nikon, Japan). GFAP- and NeuN-positive cells were magnified 20× and counted using semi-quantification analysis. Six views in each group were selected randomly, and the positive cells in each view were counted.

### Statistical analysis

All data were expressed as mean ± SD. Statistical analyses were performed using SPSS Statistics software (Version 22, IBM, NY, USA). One-way ANOVA followed by Tukey’s HSD *post hoc* test was performed for statistical comparisons. *p* values < 0.05 were considered indicative of significant differences between data sets, and *p* values < 0.01 were considered indicative of very significant differences between data sets.

## Results

### Fluorescence labeling of chitosan with RBITC

**Fig 1:**
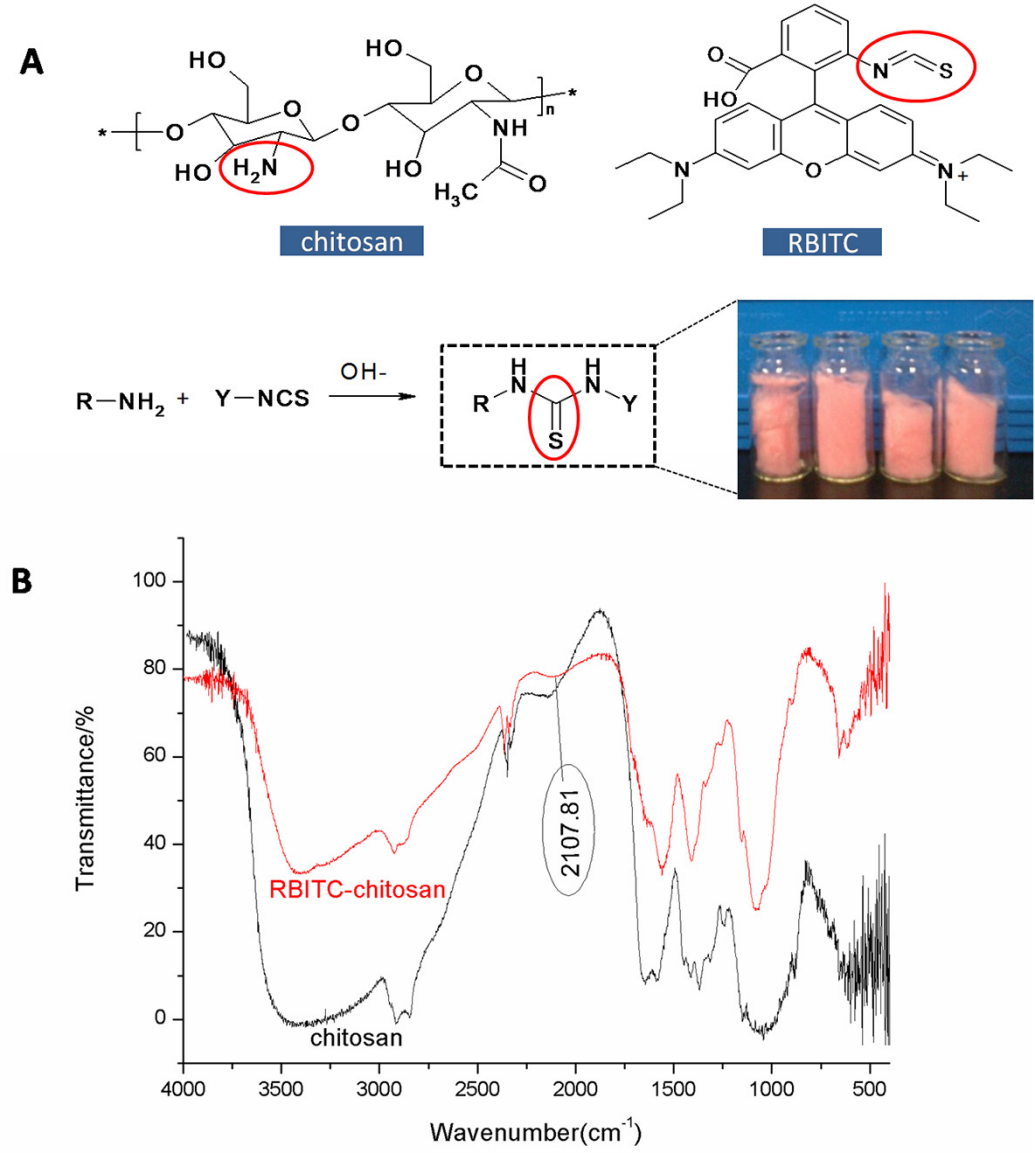
Identification of the RBITC-labeled chitosan. (A) Scheme of synthesis of the RBITC-labeled chitosan. (B) FTIR spectra of the unlabeled and RBITC-labeled chitosan.

RBITC-labeled chitosan was synthesized by the reaction between the primary amino group of chitosan and the isothiocyanate group of RBITC (Fig 1A). The RBITC-labeled chitosan after lyophilization is shown in Fig 1A. The combination of chitosan and RBITC was confirmed by FTIR spectra (Fig 1B). The appearance of the peak (N = C = S) at 2107.81 cm^-1^ in the spectrum of the RBITC-labeled chitosan compared with the chitosan spectrum proved the successful synthesis of the RBITC and chitosan.

### Characterization of TmCS-NPs and RBITC labeled TmCS-NPs

**Fig 2:**
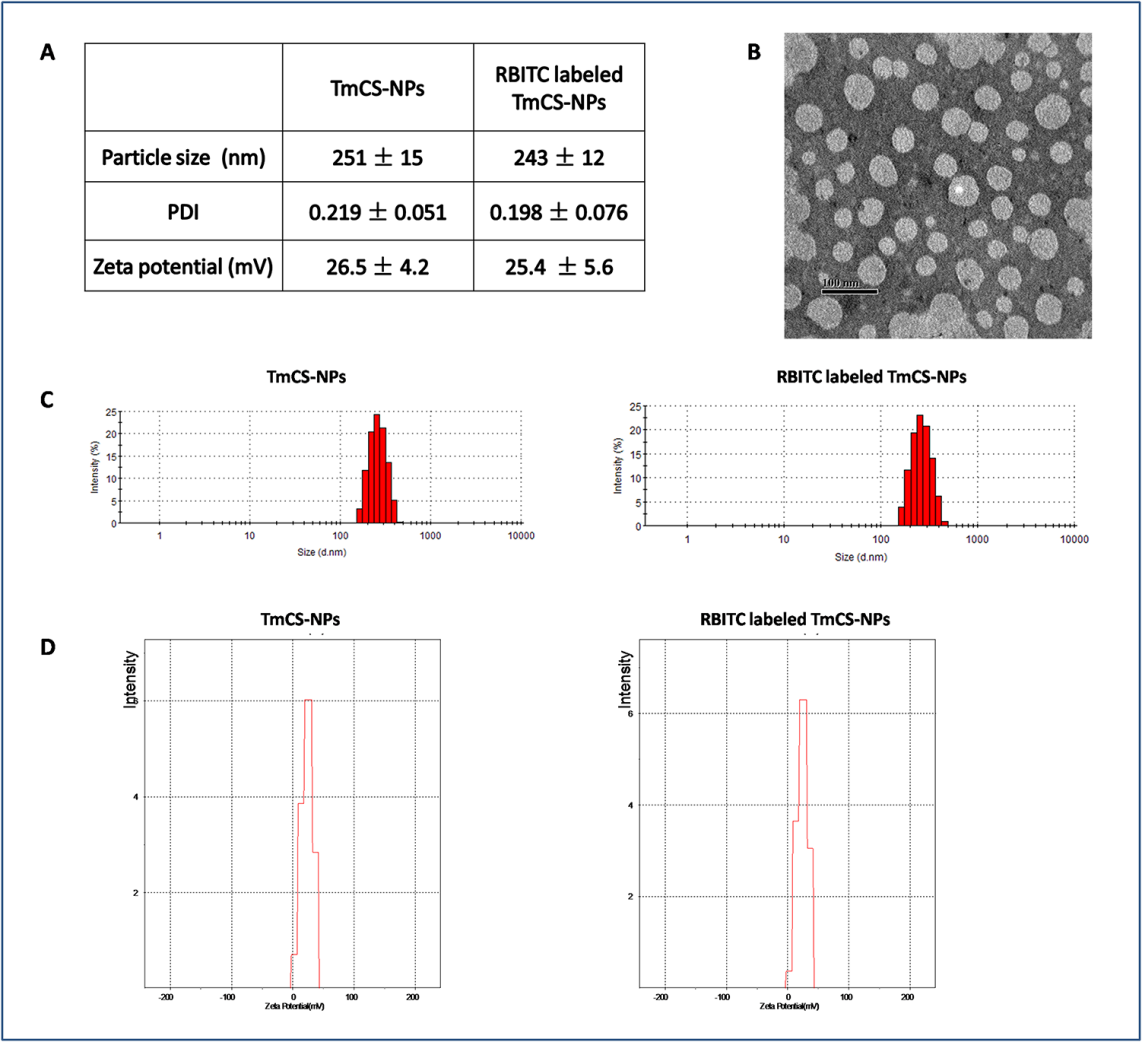
Characterization of unlabeled and RBITC-labeled TmCS-NPs. (A) Particle size, PDI, and zeta potential of the unlabeled and RBITC-labeled TmCS-NPs. (B) TEM analysis of the TmCS-NPs. (C) Particle size graphs of TmCS-NPs and RBITC-labeled TmCS-NPs. (D) Zeta potential graphs of TmCS-NPs and RBITC-labeled TmCS-NPs.

TmCS-NPs and RBITC-labeled TmCS-NPs were successfully prepared by ionic cross-linking method. The NPs prepared were characterized by dynamic light scattering (DLS) and TEM. The physicochemical properties of the NPs are summarized in Fig 2A. The morphology of the NPs observed by TEM (Fig 2B) shows that both NPs are uniformly-spherical in shape. The particle size and zeta-potentical of TmCS-NPs were 251 ± 15 nm and 26.5 ± 4.2 mV, respectively. The corresponding values for the RBITC-labeled TmCS-NPs were 243 ± 12 nm and 25.4 ± 5.6 mV. Thus, labeling with RBITC did not remarkably alter the physicochemical properties of the TmCS-NPs.

### Brain localization and accumulation

**Fig 3:**
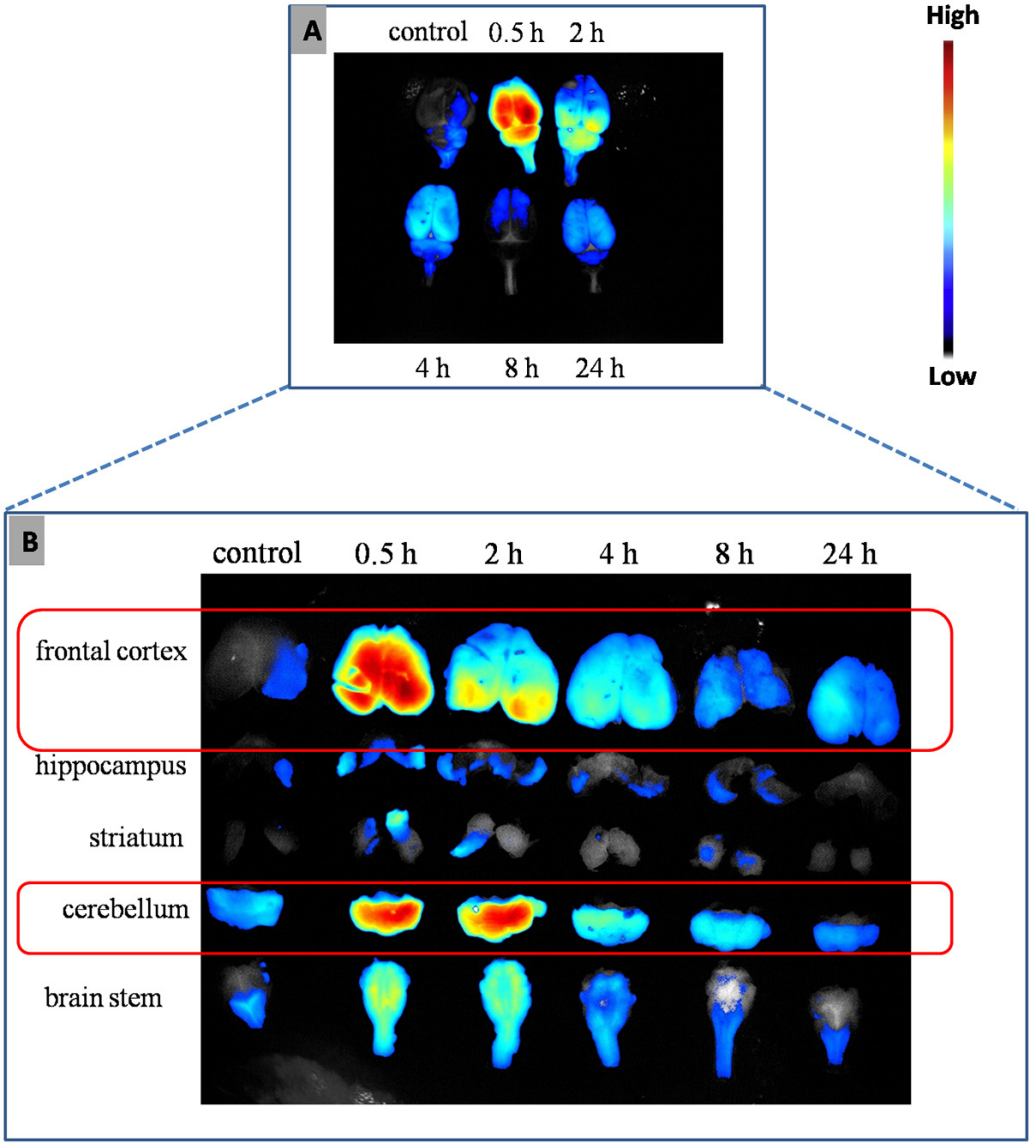
(A) Ex-vivo fluorescence images of the brains with RBITC-labeled TmCS-NPs after systemic injection at 0.5, 2, 4, 8, and 24 h. (B) Fluorescence photographs of the sub-brain regions separated from the brains above at 0.5, 2, 4, 8, and 24 h.

In order to investigate the neurotoxicity of TmCS-NPs, we should first determine their fate in the brain. In this study, intravenous injection of the RBITC-labeled TmCS-NPs resulted in a significant increase of the TmCS-NPs in the brain at each time point. And at 0.5 h after injection, TmCS-NPs widely distributed in the brain. Moreover, the quantity of the TmCS-NPs in the brain decreased as time progressed. Fluorescence signals were still detected in the brain at 24 h post-injection (Fig 3). The fluorescence signals of the sub-brain regions were evaluated to determine the location of the TmCS-NPs. The TmCS-NPs levels in the sub-brain regions were ranked in order of decreasing quantity: frontal cortex > cerebellum > brain stem > hippocampus > striatum (Fig 3). Therefore, damages in the frontal cortex and cerebellum were examined in the following study.

### Oxidative-stress-related biomarker assay and body weight change

**Fig 4:**
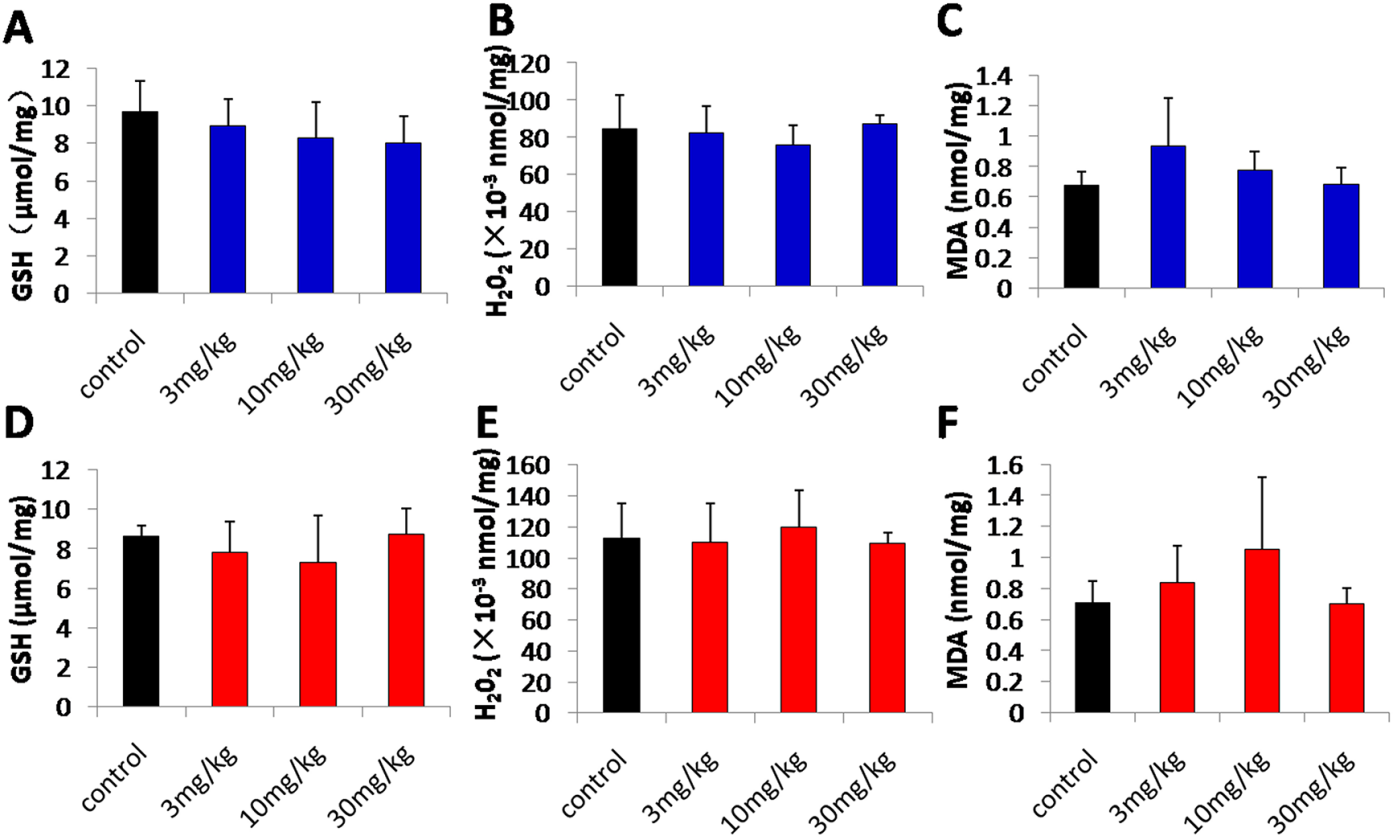
Changes in the GSH, H_2_O_2_, and MDA levels in the frontal cortex and cerebellum of rats (n = 6, six rats per group) intravenously injected with physiological saline (control) and TmCS-NPs (3, 10, and 30 mg/kg) for 7 d. (A, B, and C) GSH, H_2_O_2_, and MDA levels in the frontal cortex, respectively; (D, E, and F) GSH, H_2_O_2_, and MDA levels in the cerebellum, respectively. *p < 0.05 when compared with the control group.

**Fig 5:**
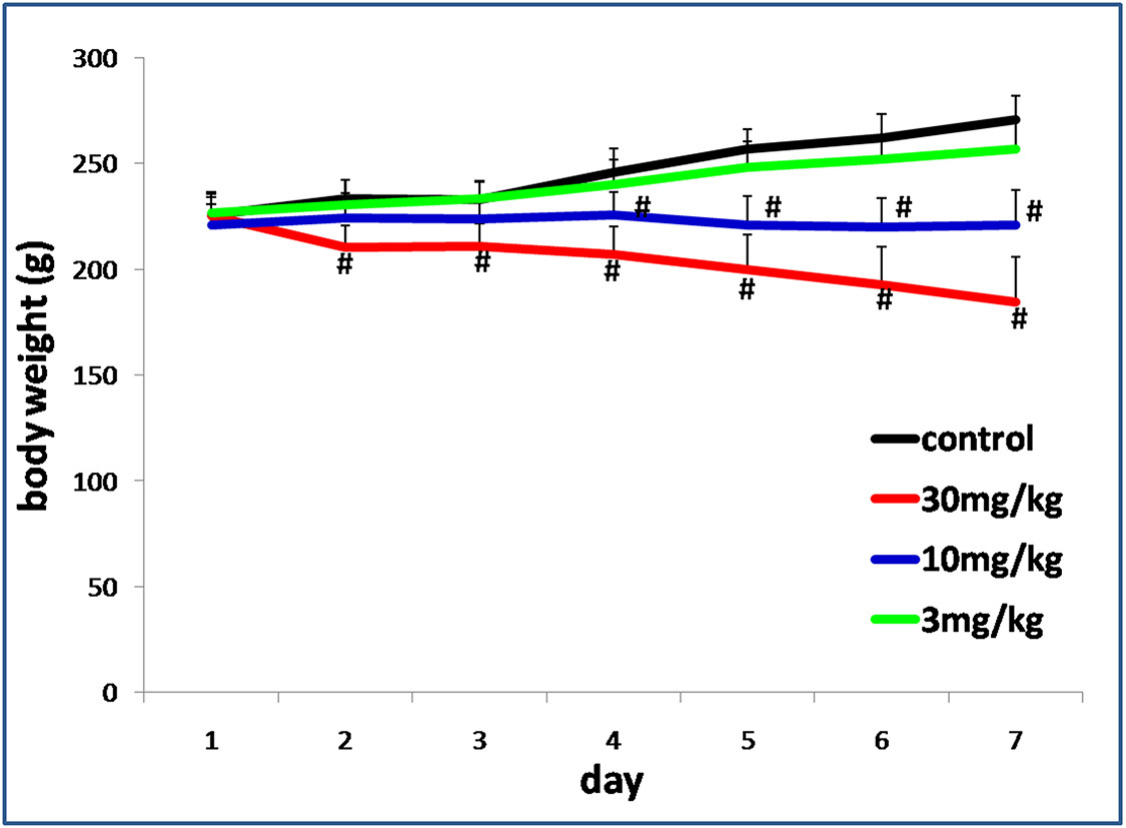
Body weight changes in rats (n = 6, six rats per group) intravenously injected with physiological saline (control) and TmCS-NPs (3, 10, and 30 mg/kg) for 7 d. *p < 0.05 when compared with the control group. #p < 0.01 when compared with the control group.

GSH activity and levels of H_2_O_2_ and MDA in the frontal cortex and cerebellum were measured to determine whether exposure of TmCS-NPs through the tail vein was the cause of oxidative stress. GSH, an important antioxidant, can prevent cellular damage induced by reactive oxygen species, such as free radicals and peroxides. GSH depletion is one of the crucial clues in cellular toxicity. H_2_O_2_ is a toxic byproduct of aerobic metabolism. It can activate the transcription factor, NF-κB, and is involved in cellular apoptosis. H_2_O_2_-related signal pathways play a crucial role in many neurodegenerative diseases. MDA is the end product of lipid peroxidation, which is toxic to body tissues. GSH, H_2_O_2,_ and MDA are the biomarkers of oxidative stress. The results of these biomarker assays showed that the TmCS-NPs-exposed groups do not exhibit significant oxidative stress damage compared with the control group (Fig 4). However, after exposure to TmCS-NPs, the body weight of the rats decreased dose-dependently compared with those in the control group (Fig 5), which indicates possible injury. Thus, a more detailed nervous system associated assessment should be conducted.

### Histopathological examination

**Fig 6:**
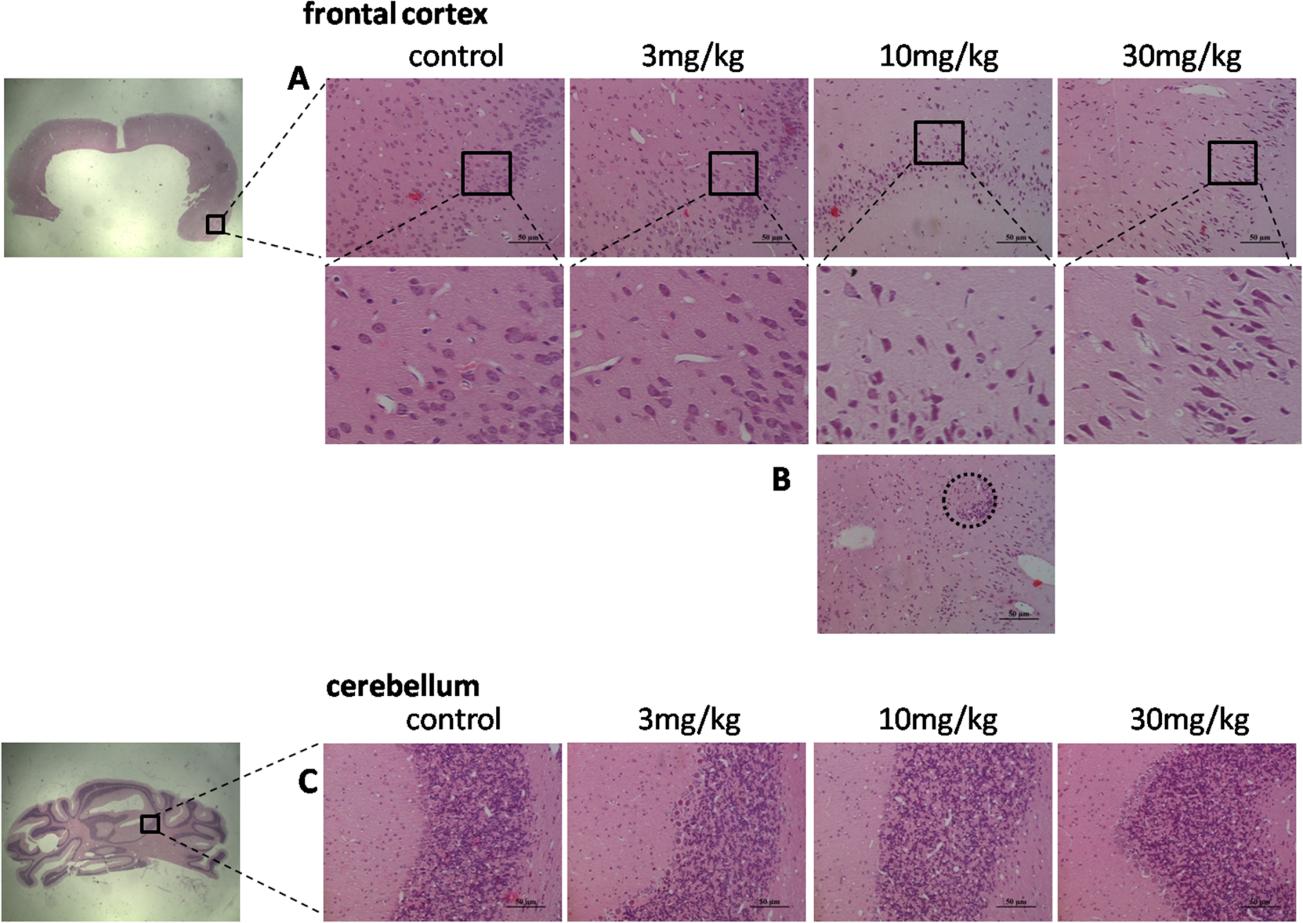
Histopathology of the rat brain tissue after intravenous injection with physiological saline (control) and TmCS-NPs (3, 10, and 30 mg/kg) for 7 d. (A) Frontal cortex region; (B) Frontal cortex region of the middle-dose group. (C) Cerebellum region.

The frontal cortex and cerebellum sections were stained with H&E to investigate the neuronal injury induced by TmCS-NPs, and the results are presented in Fig 6. For the frontal cortex sections, the apoptosis neurons in the TmCS-NPs-exposed groups in a certain area were observed. The morphology of some of the neurons changed to spindle compared with the control group (Fig 6A). This kind of pathological change became increasingly evident as the dosage of administered TmCS-NPs increased. Moreover, a lymph node was formed in the middle-dose group (10 mg/kg) as demonstrated in Fig 6B, which indicates an inflammatory response. For the cerebellum sections, the TmCS-NPs-exposed group showed no significant change in the histology of the granular layers, molecular layers, and Purkinje layers compared with the control group (Fig 6C). Thus, exposure to TmCS-NPs might exert slight toxicity to the frontal cortex but may have no effect on the cerebellum in terms of histomorphology. Brain is crucial for the maintenance of bodily function, but it is also very vulnerable that any slight inflammatory response may lead to disorders.

### Immunohistological staining of GFAP and NeuN

**Fig 7:**
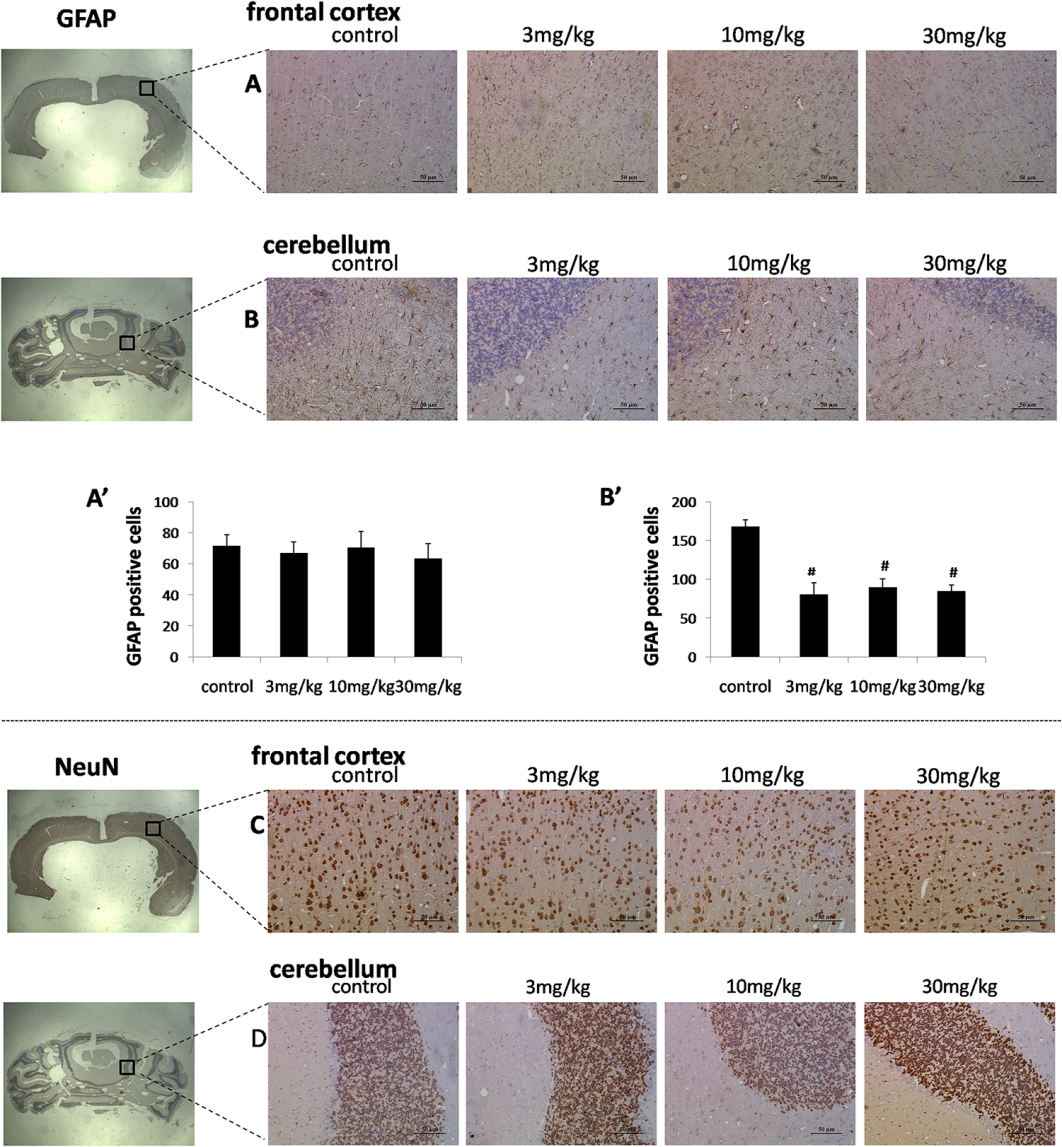
Immunohistological staining of the GFAP and NeuN of the rat brain tissue after intravenous injection with physiological saline (control) and TmCS-NPs (3, 10, and 30 mg/kg) for 7 d. (A) GFAP expression in the frontal cortex region; (A’) Semi-quantification analysis of the GFAP expression in the frontal cortex. (B) GFAP expression in the cerebellum region; (B’) Semi-quantification analysis of the GFAP expression in the cerebellum. (C) NeuN expression in the frontal cortex region; and (D) NeuN expression in the cerebellum region. *p < 0.05 when compared with the control group, #p < 0.01 when compared with the control group.

GFAP and NeuN immunoreactivity were performed in the frontal cortex and cerebellum, as shown in Fig 7. After exposure to TmCS-NPs, no significant difference in the number of NeuN-positive cells was observed in the frontal cortex or the cerebellum compared with the control group (Fig 7C and 7D). No apparent change was noted in the number of GFAP-positive cells in the frontal cortex (Fig 7A and 7A’). However, when the rats were exposed to the different doses of TmCS-NPs for seven consecutive days, a significant decrease (P < 0.01) in the number of GFAP-positive cells in the cerebellum was observed compared with those of the control group. However, the results obtained from the three different dose groups showed no significant change (Fig 7B and 7B’).

## Discussion

The first and most important step in evaluating the neurotoxicity of TmCS-NPs is to determine their fate in the brain. RBITC, a fluorescent marker, was conjugated with TmCS-NPs to detect the biodistribution of the nanoparticles in the *in vivo* rat model. After labeled with RBITC, the physicochemical properties of TmCS-NPs did not show any significant change (Fig 2A). Fig 3 shows that the frontal cortex and cerebellum appeared to be the two major accumulation regions after injection of TmCS-NPs via the tail vein. The biodistribution of TmCS-NPs in the sub-brain regions has been seldom examined. Functionalized 115 nm gold nanoparticles has been reported to mainly accumulate in the hippocampus, thalamus, hypothalamus, and cerebral cortex[25]. Another study has shown that SiO_2_ nanoparticles at a size of 156 nm in physiological saline mostly accumulated in the hippocampus and striatum[26]. However, the mechanism of the biodistribution of TmCS-NPs in the sub-brain regions is ambiguous.

Several psychiatric diseases, such as depression, schizophrenia, and obsessive-compulsive disorder, have been associated with damage of the frontal cortex[27]. And damage to the cerebellum does not cause intellectual impairment or paralysis, but it contributes to the degradation of movement, intention tremor, dysdiadochokinesia, and deficits in motor learning[28]. Therefore, whether the exposure to TmCS-NPs would induce potential injuries in the frontal cortex and cerebellum should be determined.

The primary mechanism of nanoparticle toxicity involves the production of reactive oxygen species (ROS) and free radicals. It may result in oxidative stress reactions that lead to inflammation and then cause pathology changes[18]. No obvious oxidative stress reaction in the frontal cortex or cerebellum was observed after exposure to TmCS-NPs (Fig 4). However, the middle-dose group lost weight on day 4 prior to injection of the TmCS-NP suspension, and the high-dose group lost weight 1 d post-injection (Fig 5). The body weight changes might be due to a systemic effect. Future studies should measure food consumption. However, the connection between the body weight loss and the neurotoxicity of TmCS-NPs should not be ignored. Zhang et al.[29] demonstrated that silver nanoparticles decreased the body weight and locomotor activity in adult male rats. In other words, after exposure to silver nanoparticles, rats elicited apparent neurotoxicity, such as locomotor acitivity disorders, also displayed obvious decrease in the body weight. Though there are no sufficient proofs elucidating the connection between body weight changes and neurotoxicity, potential neurotoxicity should not be ignored in rats with significant body weight decease. Thus, subtle assessments should be carried to explore the potential neurotoxicity of TmCS-NPs, such as histopathological examination, morphological structure, and number changes of neurocytes, among others.

The occurrence of apoptosis of neurons and slight inflammatory reaction in the tissue section without evident changes in oxidative-stress-related biomarkers should be explored. The weak reaction in oxidative stress and inflammation probably cannot be measured quantitatively by associated kits because of the limit of detection, but they could be detected in slices.

A significant reduction in the GFAP expression in the cerebellum white matter of the three groups was observed, but not in a dose-dependent manner (Fig 7B’), which suggested the occurrence of degenerative changes in the cerebellum after exposure to TmCS-NPs. An upregulated GFAP expression generally indicates activated astrocytes that might induce brain damage[30–32]. This study demonstrated an apparent downregulation the GFAP expression in the cerebellum after exposure to TmCS-NPs. Downregulated GFAP expression has been associated with Down syndrome, schizophrenia, bipolar disorder, Wernicke’s encephalopathy, and depression[33–35]. Significant dose-dependent downregulation of GFAP expression in the cerebellum compared with the control group has been observed after lifelong alcohol consumption[36]. The mechanisms by which chronic exposure to alcohol alters GFAP expression are unknown, but the changes may have been caused by the direct interference of alcohol with the GFAP gene transcription. The mechanism of TmCS-NPs-induced GFAP alterations may be similar to that of alcohol. Whether this change in the cerebellum, which was induced by TmCS-NPs, would cause structural and functional changes in the brain needs to be further explored.

## Conclusion

Here we reported, for the first time, that TmCS-NPs with a size of 240 nm mostly accumulated in the frontal cortex and cerebellum. Further, the quantity of TmCS-NPs in these two regions decreased over time. Fluorescence signals were still observed in the brain at 24 h post-injection. After 7 d intravenous injection with TmCS-NPs, the body weight of rats remarkably decreased dose-dependently compared with those of the control group. In addition, dose-dependent neuron apoptosis and slight inflammatory response in the frontal cortex, and downregulation of GFAP expression in the cerebellum induced by 7 d exposure to TmCS-NPs exposure were detected. These phenomena indicate the potential neurotoxicity of TmCS-NPs. Results from this study can be used in future investigations on the role of TmCS-NPs as a brain delivery carrier. A reference is thus provided for dose selection when TmCS-NPs are used as a carrier for brain disorders. However, more extensive and in-depth examinations should be considered carefully in future studies to better understand the toxicity of TmCS-NPs.
